# Predicting Remission in Schizophrenia Using Machine Learning—Assessing the Impact of Sample Size and Predictor Overinclusion

**DOI:** 10.1111/acps.70037

**Published:** 2025-09-10

**Authors:** Fredrik Hieronymus, Magnus Hieronymus, Axel Sjöstedt, Staffan Nilsson, Jakob Näslund, Alexander Lisinski, Søren Dinesen Østergaard

**Affiliations:** ^1^ Institute of Neuroscience and Physiology University of Gothenburg Gothenburg Sweden; ^2^ Department of Clinical Medicine Aarhus University Aarhus Denmark; ^3^ Swedish Meteorological and Hydrological Institute Norrköping Sweden; ^4^ Institute of Laboratory Medicine University of Gothenburg Gothenburg Sweden; ^5^ Department of Affective Disorders Aarhus University Hospital—Psychiatry Aarhus Denmark

**Keywords:** item‐level analysis, machine learning, schizoaffective disorder, schizophrenia, supervised learning, symptom remission, symptom‐level analysis

## Abstract

**Introduction:**

Machine learning studies sometimes include a high number of predictors relative to the number of training cases. This increases the risk of overfitting and poor generalizability. A recent study hypothesized that between‐trial heterogeneity precluded generalizable outcome prediction in schizophrenia from being achieved. However, an alternative explanation is that predictor overinclusion might explain the low generalizability in that analysis.

**Methods:**

Positive and Negative Syndrome Scale (PANSS) item‐data, age, sex, and treatment allocation (antipsychotic/placebo) from 18 placebo‐controlled trials of risperidone and paliperidone, in schizophrenia or schizoaffective disorder, were used as predictors for training five supervised learning models to predict symptom remission after 4 weeks of treatment. Sensitivity analyses varying the number of training cases and including simulated uninformative predictors were conducted to assess model performance, as were analyses on simulated data.

**Results:**

Better‐than‐chance predictions could be achieved for all models using as few as 384 training cases (BAC 0.60, SD 0.035 for an ensemble model). Model performance increased with the number of training cases (*n* = 4384, BAC 0.63, SD 0.041) and was higher when validated on a set of unseen trials without placebo controls (*n* = 1508, BAC 0.68, SD 0.013). Predictive performance was substantially decreased by including simulated uninformative predictors. Analyses of simulated data suggest that considerably larger sample sizes than commonly used might be required to effectively separate weakly informative from uninformative predictors.

**Conclusion:**

Supervised learning models can generate better‐than‐chance predictions in schizophrenia from small datasets, but this requires that not too many uninformative predictors are included. Since highly predictive models have not yet been established for schizophrenia—and since strong linear predictors are easy to identify—commonly collected clinical trial data likely do not contain predictors with strong linear relations to clinically relevant outcomes. If correct, future machine learning analyses should focus on maximizing the probability of identifying weakly predictive features.

**Summary**

- Significant outcomes
  ○ Better‐than‐chance predictions of symptom remission in schizophrenia could be achieved using relatively small sets of training data (*n* = 384).
  ○ Including simulated uninformative predictors, in addition to informative ones, substantially decreased model performance compared to models where such predictors were not included.
  ○ Analyses of simulated data suggest that strong linear relations are easy to detect also when using small data sets. That such relations have not yet been identified suggests that they are unlikely to exist, and that effort should be focused on maximizing the probability of detecting weak and/or complex relationships.
- Limitations
  ○ This analysis focuses on illustrating the detrimental effects of including too many predictors. Several design choices, for example, only analyzing one treatment outcome, are suboptimal had the goal been to provide the best possible model for predicting treatment outcomes in schizophrenia.
  ○ Simulation analyses are limited to two ML models (elastic net and linear regression) and linear predictors of different strengths. They hence cannot inform on the performance of other ML models, or on model performance in detecting non‐linear relationships.

## 1 Introduction

While individualized treatment based on tumor and patient characteristics is becoming a reality for several cancers [1, 2], there are few parallels in other fields of medicine [3]. For psychiatry, no major clinical breakthroughs have occurred despite considerable interest in precision psychiatry [4, 5, 6]. In line with this, a recent study by Chekroud and colleagues reported that two supervised learning algorithms (elastic net and random forest) failed to produce generalizable predictions of symptom remission when trained on approximately 1500 cases. They hypothesized that this failure might be due to considerable between‐trial heterogeneity [7].

Supervised learning models are a subset of machine learning (ML) models that are commonly employed for clinical prediction [6]. In supervised learning, the outcome of interest—for example, will this patient remit after 4 weeks of treatment—is known, and the models are tasked with approximating the function that best maps the predictors to the outcome. Provided with reasonable parameters regarding, for example, validation strategy and hyperparameter tuning, supervised learning models tend to perform well at detecting also weak and complex predictor‐outcome relations [6]. However, they perform less well when the number of studied predictors (*p*) is large relative to the number of training cases (*n*), that is, on high‐dimensional data with few independent observations. One well‐known observation related to this—sometimes called the “peaking phenomenon”—is that model performance often peaks at an intermediate feature set size and then declines as more predictors are added [8, 9, 10]. Since the study by Chekroud et al. [7] utilized a large number of predictors (*p* = 217 for elastic net; *p* = 137 for random forest) relative to their sample size (*n* = 1032 to 1414 participants; in leave‐one‐study‐out analysis) an alternative explanation for the poor observed predictivity is that the models performed poorly due to the peaking phenomenon.

## 2 Aims of the Study

In this study we utilize data from the same provider (Yale University Open Data Access, YODA) [11] as Chekroud and co‐workers to show (i) that the peaking phenomenon is a sufficient explanation for the poor predictivity observed in their analysis and (ii) that models which include fewer predictors can be more accurate also when trained on fewer cases. We further argue—using simulated data—that issues related to the peaking phenomenon are likely widespread.

## 3 Materials and Methods

### 3.1 Data Acquisition

Patient‐level data for all industry‐sponsored, acute‐phase, placebo‐controlled trials of risperidone and/or paliperidone in schizophrenia were requested via the YODA portal [11]. Access to patient‐level data was provided by Johnson & Johnson and YODA for these 19 studies. One study (RIS‐USA‐1/Study 201) did not use the Positive and Negative Syndrome Scale (PANSS) [12] for symptom rating and could not be included. The remaining placebo‐controlled trials constitute the train‐and‐test dataset.

We also requested patient‐level data for three acute‐phase trials of risperidone and/or paliperidone in schizophrenia that did not include a placebo control. These actively controlled trials were solely used for model validation and are referred to as the extender set.

Data accuracy was verified against study reports from YODA, as well as against reports from the United States Food and Drug Administration [13, 14, 15, 16, 17, 18, 19], the European Medicines Agency [20] and ClinicalTrials.gov [21, 22, 23]. No major inconsistencies were found. We have previously used this dataset to assess the utility of unidimensional PANSS subscales [24, 25].

### 3.2 Statistical Analysis

R version 4.3.0 (via the YODA Remote Desktop Environment) was used for analyses of trial data, and R version 4.3.2 was used for analyses of simulated data. The outcome for all analyses was cross‐sectional symptom remission after 4 weeks of treatment. In line with the criteria established by the Remission in Schizophrenia Working Group [26], this was defined as achieving a score of ≤ 3 (mild) on eight core PANSS items by week 4, but without the 6‐month duration requirement. Only patients with a week 4—or week 5 in studies that lacked an evaluation at week 4—PANSS assessment were included.

Supervised learning models were trained using the Classification and Regression Training (caret) package in R (version 6.0–94), with ensemble models trained using the caretEnsemble package (version 2.0.3). All models maximized the area under the receiver operating curve (AUC‐ROC) and used 10‐fold cross‐validation. Balanced accuracy, BAC—the mean of sensitivity and specificity—is reported for all analyses.

### 3.3 Analyses of Schizophrenia Trial Data

To assess the impact of including a large number of predictors—many of which are likely to be uninformative—we first replicated the analyses by Chekroud and colleagues with two modifications: (i) rather than using leave‐one‐study‐out methodology, we subsampled training and test sets corresponding in size to the by‐study splits used by Chekroud and co‐workers from the placebo‐controlled trials and (ii) rather than including 217 (elastic net) or 137 (random forest) predictors, as done by Chekroud et al. we included either (a) a subset of 33 of the predictors used by Chekroud and colleagues (the 30 PANSS items at baseline, age, sex and treatment allocation [antipsychotic yes/no]) which had shown promise in previous and preliminary analyses or (b) those 33 predictors plus 184 or 104 simulated predictors known to be uninformative. These modifications were done (i) to make sure that a potential finding of poor predictive performance could not be explained by between‐study heterogeneity and (ii) since it allows for the assessment of model performance in the presence of predictors known to be uninformative. Hyperparameter tuning was done individually for each testing set; like Chekroud and co‐workers, we tested 400 elastic net and 20 random forest models. The resultant estimates were compared to the single‐study predictions reported by Chekroud et al.

We then trained an ensemble of five supervised learning models: Bagged Classification and Regression Trees (Bagged CART; caret method: treebag), elastic net (caret method: glmnet), logistic regression (caret method: glm), random forest (caret method: rf) and extreme gradient boosting trees (caret method: xgbTree) on the 33‐predictor subset. A glm was used to weigh the model predictions together (R‐package caretEnsemble). Genetic algorithms (R‐package: GA; version 3.2.3) were used for hyperparameter tuning of the xgbTree and glmnet models, and the rf model was tuned using an exhaustive grid search (see Table S1). Bagged CART and glm do not use hyperparameter tuning. Due to processing power limitations, deeper hyperparameter tuning was done separately using the full placebo‐controlled dataset and only a single hyperparameter tuning per model was explored during the different validation steps. However, all models still used 10‐fold cross‐validation. Details on the hyperparameter tuning process, including number of generations, stopping criteria, search bounds, final tunings, and so forth can be found in Table S1.

### 3.4 Assessing the Impact of Training Set Size

Ensemble models were trained on Monte Carlo subsampled subsets from the placebo‐controlled dataset. These ranged in size from 384 to 4384 cases. Fifty subsampled training sets were constructed for each training set size. For each such training set, 100 subsampled or bootstrapped test sets of 250 cases were drawn from the placebo‐controlled cases not used for model training.

### 3.5 Validation on Unseen Data From Active‐Control Trials

Ensemble model performance was similarly validated against data withheld from both hyperparameter tuning and model training, that is, with data from the three active‐controlled trials used as the test set.

### 3.6 Leave‐One‐Study‐Out Validation

To assess cross‐trial heterogeneity, we conducted leave‐one‐study‐out analyses in which 17 trials from the train‐and‐test set were used for model training and the 18th was reserved for testing. To compare the results to what might be expected from chance, 50 subsampled training sets corresponding in size to withholding the smallest (*n* = 4536) and largest (*n* = 4163) studies were created. Predictions for the withheld cases were used to create 95% confidence intervals (CIs) to which the leave‐one‐study‐out predictions could be compared.

### 3.7 Analyses of Simulated Data

To illustrate the issues with data including weak and uninformative predictors, we simulated 3300 datasets ranging in size from 250 to 25,000 cases, with different ratios of informative to uninformative predictors (1:3 or 1:9) and where predictor strength varied (from *r* = 0.05 to 0.50). The simulated data enabled us to calculate a theoretical limit on the predictive accuracy that could be achieved in each dataset. This was then compared to the performance achieved by an elastic net model and a logistic regression model, respectively, trained on 80% of the data. 400 hyperparameter tunings were explored per elastic net model.

For further details on the methodology, please refer to the Supporting Information.

## 4 Results

As shown in Table 1, the placebo‐controlled dataset consists of 18 trials in which 6689 participants (*n*‐range: 114 to 651) were randomized and where 4634 individuals had outcome data available at week four. The four‐week retention rate was 61% for those randomized to placebo (1097 of 1796) and 72.2% for those randomized to antipsychotics (3537 of 4893). For those with outcome data, symptom remission after 4 weeks was attained by 36.4% of those receiving antipsychotics (1286) and by 31.1% of those receiving placebo (341). The actively controlled set consists of 3 trials in which 1725 participants were randomized and where 1508 individuals (87.4%) had outcome data available after 4 weeks of treatment. Symptom remission was attained by 38.8% (585) of those with available outcome data. Baseline scores for all PANSS items are provided in Table S2.

**TABLE 1:** Included studies.

Dataset	Protocol	*N* randomized	# 4‐week completers (%)	# remission (%)	# placebo (%)	# male (%)	Age mean (SD)
Train‐and‐test	PALM‐JPN‐4^b^ (#1)	324	222 (69)	63 (28)	106 (48)	127 (57)	45.3 (13.2)
	R076477‐PSZ‐3001 (#2)^a^	201	156 (78)	72 (46)	32 (21)	93 (60)	15.2 (1.5)
	R076477‐SCA‐3001 (#3)	314	215 (68)	150 (70)	63 (29)	142 (66)	37.0 (9.9)
	R076477‐SCA‐3002 (#4)	311	197 (63)	102 (52)	53 (27)	106 (54)	37.6 (9.0)
	R076477‐SCH‐3015 (#5)^a^	398	323 (81)	153 (47)	60 (19)	207 (64)	35.8 (10.8)
	R076477‐SCH‐302 (#6)^a^	114	98 (86)	29 (30)	30 (31)	27 (28)	69.5 (4.5)
	R076477‐SCH‐303 (#7)^a^	629	471 (75)	163 (35)	74 (16)	241 (51)	37.0 (11.3)
	R076477‐SCH‐304 (#8)	449	231 (51)	90 (39)	48 (21)	169 (73)	42.3 (10.7)
	R076477‐SCH‐305 (#9)^a^	617	427 (69)	131 (31)	66 (15)	286 (67)	37.3 (10.7)
	R076477‐SCH‐4012 (#10)	201	124 (62)	43 (35)	39 (31)	85 (69)	40.8 (12.3)
	R092670‐PSY‐3003^b^ (#11)	356	230 (65)	89 (39)	81 (35)	156 (68)	39.2 (10.6)
	R092670‐PSY‐3004^b^ (#12)	516	371 (72)	126 (34)	84 (23)	245 (66)	39.8 (11.4)
	R092670‐PSY‐3007^b^ (#13)	651	469 (72)	123 (26)	107 (23)	303 (65)	39.1 (10.8)
	R092670‐SCH‐201 (#14)	243	172 (71)	54 (31)	49 (28)	111 (65)	39.1 (10.5)
	RIS‐INT‐3 (#15)	520	336 (65)	63 (19)	44 (13)	284 (85)	37.6 (10.4)
	SCH‐302 (#16)	157	137 (87)	35 (26)	47 (34)	88 (64)	15.7 (1.3)
	USA‐121 (#17)	443	294 (66)	79 (27)	62 (21)	205 (70)	39.1 (9.6)
	USA‐72 (#18)	245	161 (66)	62 (39)	52 (32)	129 (80)	37.7 (9.1)
Extender	USA‐231	279	224 (80)	81 (36)	0 (0)	130 (58)	15.2 (1.9)
	R092670‐PSY‐3006^b^	1218	1070 (88)	421 (39)	0 (0)	610 (57)	38.9 (12.0)
	R076477‐PSZ‐3003	228	214 (94)	83 (39)	0 (0)	139 (65)	15.3 (1.5)

*Note:* Protocol numbers in parentheses are used to reference individual studies in Figures 1 and 2.

^a^ Study was included in the analysis by Chekroud and co‐workers.

^b^ Study did not have an evaluation scheduled after 4 weeks of treatment, data from week 5 was used instead.

**FIGURE 1:**
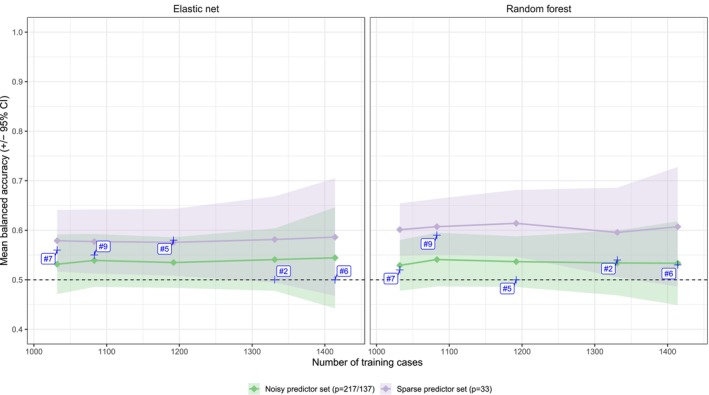
Four‐week remission prediction: Comparison between models including or not including simulated uninformative predictors. Solid lines represent mean subsampled BAC and ribbons give 95% CIs for 50 Monte Carlo subsamples (25 for random forest) of the same train and test set sizes as used in the analysis by Chekroud and colleagues. Test sets were bootstrapped 100 times for each subsampled training set. Each number (#2, #5, #6, #7 and #9) represents one of the single‐study accuracies reported by Chekroud and co‐workers (see Table 1).

Figure 1 shows the results of models predicting four‐week symptom remission in schizophrenia using a moderate amount of training data (*n* = 1032 to 1414) and including (green) or not including (purple) simulated uninformative predictors. BAC was substantially decreased by including uninformative predictors. The impact was larger for random forest (average BAC 0.61 to 0.53; SD‐range 0.026 to 0.062) than for elastic net (average BAC 0.58 to 0.54; SD‐range 0.026 to 0.061). The confidence intervals (CIs) for the prediction estimates increased with training set size, reflecting increased variability as the test set decreased in size. All single study estimates from Chekroud et al. [7] for the elastic net model (Figure 1, left panel) lie within the 95% CIs, regardless of whether uninformative predictors were included or not, indicating that low out‐sample predictivity is not contingent on between‐study heterogeneity. For the random forest model (Figure 1, right panel), all single‐study estimates lie within the 95% CI for models including simulated predictors, and two out of five were worse than expected compared to models not including simulated predictors.

**FIGURE 2:**
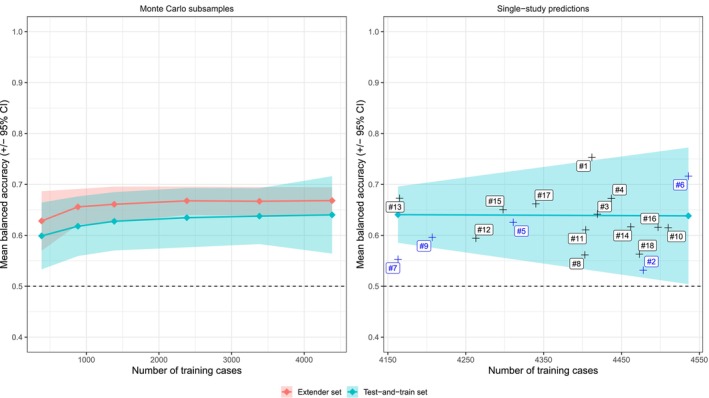
Four‐week remission prediction: Ensemble predictions of subsampled test data, active‐control test data, and single‐study data. The left panel gives mean and 95% CI BAC for subsampled training sets ranging in size between 384 and 4384 cases. The right panel shows the same for subsampled and single‐study BAC for leave‐one‐study‐out analyses. Each number #1 to #18 represents one study in the train‐and‐test set, with blue text indicating a trial included in the analysis by Chekroud and co‐workers (see Table 1 for mapping).

Figure 2 shows the performance of an ensemble model predicting symptom remission after 4 weeks of treatment using different training set sizes. BAC for Monte Carlo subsampled test sets increased from 0.60 (SD 0.035) to 0.63 (SD 0.041) as the number of cases used for training increased from 384 to 4384. For the actively controlled trials (the extender set), BAC correspondingly increased from 0.63 (SD 0.035) to 0.68 (0.013) (Figure 2, left panel). The 95% CIs did not overlap with 0.50 (i.e., the performance of a random classifier) at any point. In leave‐one‐study‐out analyses (Figure 2, right panel), 16 out of 18 single‐study estimates of antipsychotic remission prediction were within the 95% CIs for Monte Carlo subsampled sets of the corresponding size.

Each individual model included in the ensemble also yielded better‐than‐chance predictions, i.e., the 95% CIs did not overlap 0.50 for any individual model. Extreme gradient boosting (BAC‐range in the placebo‐controlled trials: 0.61 to 0.63) was the best performing individual model and elastic net (BAC‐range in the placebo‐controlled trials: 0.58 to 0.59) the worst. Further metrics for all individual models, and the ensemble model, are provided in Figures S1–S5 and in Tables S3–S8.

**FIGURE 3:**
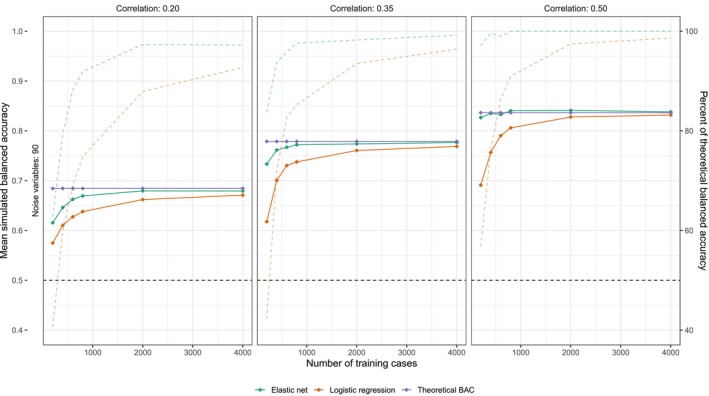
Performance of elastic net and logistic regression in simulated data with 10 strong and 90 uninformative predictors. The purple lines show theoretical BAC for the sum of all informative predictors. The orange lines show mean test‐set BAC achieved by a logistic regression model, and the green lines show the same for an elastic net model (means are averaged over 50 simulations per training set size). The dashed lines show the percent of theoretical BAC that each model (orange and green) achieves.

Figure 3 shows the performance of elastic net and logistic regression models in simulated data with strong predictors (*r* = 0.20 to 0.50) and strong noise (10 informative to 90 uninformative variables). Under these conditions, elastic net models clearly outperform logistic regression and, using 400 cases for training, achieve 79% (*r* = 0.20), 94% (*r* = 0.35) and 100% (*r* = 0.50) of theoretically optimal performance compared to 60%, 72%, and 76% for logistic regression.

**FIGURE 4:**
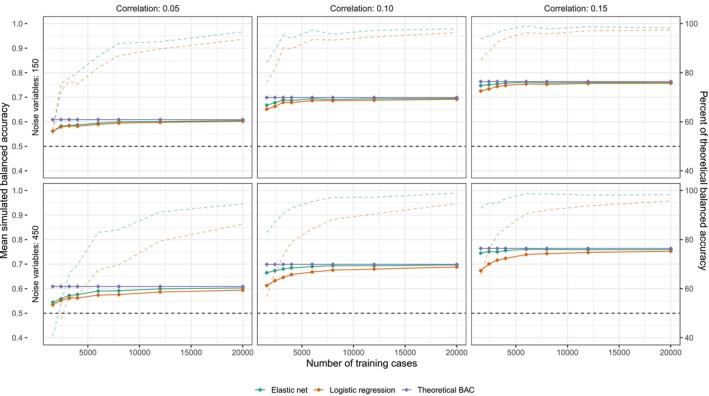
Performance of elastic net and logistic regression in simulated data with 50 weak and 150 or 450 uninformative predictors. The purple lines show theoretical BAC for the sum of all informative predictors (*p* = 50). The orange lines show test‐set BAC for a logistic regression model, and the green lines show the same for an elastic net model (means are averaged over 50 simulations per training set size). The dashed lines show the percent of theoretical BAC that each model (orange and green) achieves.

The performance advantage of elastic net diminishes as predictor strength decreases (Figure 4). Using the same signal‐to‐noise ratio (50 informative to 450 uninformative variables), 1600 cases for training, and a predictor‐outcome correlation of 0.15, elastic net achieves 93% of the theoretically optimal BAC (logistic regression: 66%). With a predictor‐outcome correlation of 0.05, however, elastic net (41% of theoretically optimal BAC) performs about as poorly as logistic regression (31% of theoretical BAC). Increasing the signal‐to‐noise ratio (50 informative to 150 uninformative predictors) improves the performance of both elastic net (57% of theoretical BAC) and logistic regression (57% of theoretical BAC) when 1600 cases are used for training.

## 5 Discussion

The main findings of this study are (a) that generalizable treatment outcome predictions for schizophrenia can be achieved using a small training set (*n* = 384) when a limited number of informative predictors (*p* = 33) are used (Figure 2), but b that when a high number of uninformative predictors are included—in addition to the informative ones—generalizable predictions are not achieved even using much larger training samples (*n* = 1414) (Figure 1).

That increases in model performance can be achieved by including fewer predictors might seem counterintuitive, but it is a well‐known observation often referred to as the peaking phenomenon [8, 9, 10]. Importantly, while including more predictors decreases generalizability, it often improves in‐sample performance metrics (i.e., it makes it easier for the models to overfit). Researchers working with small datasets—who might be understandably reluctant to further decrease available sample size by splitting their data into training and testing sets—and who rely on in‐sample performance metrics may thus be erroneously incentivized to maximize predictor inclusion even though this likely decreases model performance on unseen data.

A systematic review from December 2022 identified fourteen ML studies in schizophrenia [27]. Twelve of the 14 studies included fewer than 1000 subjects, and nine out of 14 studies included fewer than 500 subjects. Four of the included studies did not describe their validation process, and the review did not differentiate between metrics derived from in‐sample validation (such as K‐fold cross‐validation) or out‐sample validation. It also did not provide the number of predictors included in each model. Extrapolating from our results, it is thus, in most cases, not possible to evaluate the strength of the models studied from the systematic review or from the original publications. In the same vein, commonly used guidelines for developing prediction models, like PROBAST [28], also do not consider the issues of using in‐sample performance metrics to evaluate models with a high number of predictors relative to sample size, suggesting that these issues are underrecognized.

Since strong linear predictors are easy to detect (see Figure 3), the dearth of accurate and generalizable prediction models in psychiatry implies that there exist no strong linear relations between clinically relevant treatment outcomes and commonly studied blood markers, sociodemographic variables, symptomatology, genetic information, etc. Such variables—which are commonly collected during clinical trials—have received considerable formal and informal attention for a long time. Had strong linear relations existed, they would thus likely already have been identified [4, 5, 6]. Especially considering that not much has changed in terms of how we delineate and manage the major psychiatric disorders over the last decades. To the extent that such datasets contain informative predictor‐outcome relations, it is hence likely that those relations will be weak and/or complex [4, 5, 6]. If correct, future analyses would benefit from focusing on identifying those weak—and/or complex—features that likely do exist through a rigorous application of feature selection techniques.

Considering that potentially relevant predictors—for example, baseline symptomatology, heritability, sociodemographic factors, treatment history, clinical chemistry, blood markers, EEG findings, and so forth—likely number in the thousands, feature selection poses a considerable challenge. Even if, as in Figure 1, it is known that 184 out of 217 predictors are uninformative, the number of ways of selecting 33 variables out of 217 exceeds 10^39^. It is hence not a problem that can be brute‐forced.

While data‐driven feature selection methods [29, 30] should routinely be assessed, the importance of domain knowledge and hypothesis‐driven research efforts should also not be underestimated [31]. Case in point, the elastic net algorithm is considered especially useful when the number of predictors is high relative to sample size [32], but it still did not perform very well in many of the cases studied here (Figures 1, 2 and 4). Based on previous research, it is evident that baseline symptomatology is likely to be of importance for outcome prediction in schizophrenia [33]. It is less obvious that, for example, a panel of standard blood tests holds the same promise. Including sensitivity analyses of reasonably sized and qualitatively different predictor subsets is a hypothesis‐driven way in which exploring complex predictor spaces might be made more manageable. If nothing else, it can serve as a sanity check in that if better results are achieved by starting with a reduced feature set than with all available predictors, then the data‐driven feature selection techniques applied are obviously not performing satisfactorily. Alternatively, if comparable results are obtained with predictors that are easier to interpret (or obtain) then that too may be of value.

### 5.1 Limitations

This study has some limitations. First, it only includes data from the two antipsychotic development programs available via YODA [11]. Thanks to the collaboration between YODA and Vivli.org [34] it would now be possible to include data also from the development programs for aripiprazole, lurasidone, and olanzapine. Second, the Remote Desktop Environment provided by YODA had some limitations regarding software availability and processing power that restricted which analyses could be undertaken. Analyses of randomly subsampled predictor subsets would have been interesting to use as a benchmark, but they were unfeasible to conduct. Considering that future analyses are bound to get more computationally intensive, processing power is likely to become a limiting factor. While there are data safety concerns [35], establishing and adopting procedures for offline data use, as, for example, the National Institutes of Health have done [36], would improve the prospects for independent researchers to conduct large‐scale ML analyses. Alternatively, data sharing organizations could work towards improved platform interoperability alongside allowing researchers to purchase additional computer resources (as Vivli has done). Third, the extender data set had a slightly higher rate of symptomatic remission (38.8% vs. 35.1%), a lower rate of dropout (12.6% vs. 30.7%), and consisted of trials where there was no risk of receiving placebo. While thus demonstrating some degree of transferability, this is not an ideal dataset for validation since it systematically differs from the placebo‐controlled set. Fourth, there are several ways to make the analyses more informative with regard to predicting symptom remission in schizophrenia. We used the default threshold of 0.5 when calculating BAC. Introducing threshold optimization techniques might have further increased predictive performance. In the same vein, subjecting the full predictor set used by Chekroud and colleagues to rigorous feature selection techniques might have yielded a more informative predictor subset than the a priori, symptom‐focused one that was used. Similarly, including data on early treatment response is likely to improve predictive accuracy and may be nearly as clinically useful (e.g., for getting early guidance on treatment switching) [37, 38]. Training models to predict other clinically relevant outcomes, for example, treatment attrition and severe adverse events, is equally interesting. Not only are those outcomes relevant in themselves, but they are also not necessarily best predicted by the same features that predict symptomatic remission. Coupling models trained to predict very poor—or for that matter average—outcomes to models trained to predict good outcomes might yield overall increases in predictivity [39]. Fifth, the focus of this analysis was to demonstrate the detrimental impact that the peaking phenomenon can have on ML analyses. While generalizable predictions were achieved, it is not clear to what degree these rely on trivial features, for example, that patients who, at baseline, lie close to the symptom remission cut‐off have a higher chance of attaining remission after 4 weeks than patients who lie far from the cut‐off. Reverse‐engineering the models to understand which features are predictive is an important next step. Sixth, our analyses using subsampled data with simulated uninformative predictors (Figure 1) largely mirrored the results reported by Chekroud and colleagues (elastic net BAC 0.54; random forest BAC 0.53). While this does not show that between‐study heterogeneity did not play a role for the outcome of their analyses, it—combined with the better leave‐one‐study results observed when using more cases for training (Figure 2)—suggests that between‐study heterogeneity is not an insurmountable barrier to obtaining generalizable predictions of treatment outcomes in schizophrenia.

To conclude, we have demonstrated that supervised learning models trained using a low number of cases (*n* = 384) and predictors (*p* = 33) can outperform the same models trained on more data and including more predictors. This is not an unexpected result, but the issues associated with including a high number of predictors while relying on in‐sample performance metrics are likely underrecognized. While the present results (BAC 0.63 to 0.68) are far from being clinically useful, they are promising in that they are compatible with a situation where more training data might ultimately lead to prediction models with significant clinical utility. To give us the best chance at discovering such models, we argue that future ML analyses should focus more on identifying which variables hold usable information so that better models can be achieved iteratively. While, as shown in Figures 3 and 4, increasing the number of training cases is a straightforward way to improve signal detection, this is usually not possible. Therefore, both data‐driven and hypothesis‐driven strategies for reducing the number of included predictors should routinely be applied in future analyses.

## Author Contributions

F.H. had full access to all of the data in the study and takes responsibility for the integrity of the data and the accuracy of the data analysis. Conceptualization: F.H., M.H., and S.D.Ø. Methodology: F.H., M.H., A.S., S.N., and S.D.Ø. Data acquisition: F.H. and S.D.Ø. Data analysis: F.H. and M.H., Visualization: F.H. Supervision: F.H. and S.D.Ø. Interpretation: F.H., M.H., A.S., A.L., J.N., S.N., and S.D.Ø. writing – original draft: F.H., M.H., and S.D.Ø. Writing – substantial review and editing: F.H., M.H., A.S., A.L., J.N., S.N., and S.D.Ø.

## Conflicts of Interest

F.H. has received speaker's fees from Janssen Pharmaceuticals and H Lundbeck and has worked as a consultant for Flow Neuroscience. S.D.Ø. received the 2020 Lundbeck Foundation Young Investigator Prize. S.D.Ø. owns/has owned units of mutual funds with stock tickers DKIGI, IAIMWC, SPIC25KL, and WEKAFKI and owns/has owned units of exchange‐traded funds with stock tickers BATE, TRET, QDV5, QDVH, QDVE, SADM, IQQH, USPY, EXH2, 2B76, IS4S, OM3X, and EUNL. The remaining authors declare no conflicts of interest.

## Peer Review

The peer review history for this article is available at https://www.webofscience.com/api/gateway/wos/peer‐review/10.1111/acps.70037.

## Supporting information


**Table S1:** Hyperparameter tuning using genetic algorithms and grid search.
**Table S2:** Baseline item scores for the train‐and‐test set and the extender set.
**Table S3:** Additional metrics for the ensemble model.
**Table S4:** Additional metrics for the glm model.
**Table S5:** Additional metrics for the glmnet model.
**Table S6:** Additional metrics for the rf model.
**Table S7:**. Additional metrics for the treebag model.
**Table S8:** Additional metrics for the xgbTree model.
**Figure S1:** Four‐week remission: BAC for glm (subsampled test data, extender test data, and single‐study data).
**Figure S2:** Four‐week remission: BAC for glmnet (subsampled test data, extender test data, and single‐study data).
**Figure S3:** Four‐week remission: BAC for rf (subsampled test data, extender test data, and single‐study data).
**Figure S4:** Four‐week remission: BAC for treebag (subsampled test data, extender test data, and single‐study data).
**Figure S5:** Four‐week remission: BAC for xgbTree (subsampled test data, extender test data, and single‐study data).

## Data Availability

Data accession numbers for the 21 trials analyzed in this publication are provided in the Supporting Information. The code to reproduce all results is available at Zenodo (DOI: https://doi.org/10.5281/zenodo.10976022). The data that support the findings of this study are available from YODA. This study, carried out under YODA Project 2019–3941, used data obtained from the Yale University Open Data Access Project, which has an agreement with Janssen Research & Development, L.L.C. The data that support the findings of this study are available from YODA. Restrictions apply to the availability of these data, which were used under license for this study. Data are available from https://yoda.yale.edu/ with the permission of YODA. The interpretation and reporting of research using this data are solely the responsibility of the authors and do not necessarily represent the official views of the Yale University Open Data Access Project or Janssen Research & Development, L.L.C.
