# Supplementary material for: Predicting Remission in Schizophrenia Using Machine Learning—Assessing the Impact of Sample Size and Predictor Overinclusion
Source: Acta Psychiatr Scand. 2025 Sep 10;152(6):441–50. doi: 10.1111/acps.70037 (PMC12589857; doi:10.1111/acps.70037)
Supplement: Supplementary file 1 — Table S1: Hyperparameter tuning using genetic algorithms and grid search. Table S2: Baseline item scores for the train‐and‐test set and the extender set. Table S3: Additional metrics for the ensemble model. Table S4: Additional metrics for the glm model. Table S5: Additional metrics for the glmnet model. Table S6: Additional metrics for the rf model. Table S7:. Additional metrics for the treebag model. Table S8: Additional metrics for the xgbTree model. Figure S1: Four‐week remission: BAC for glm (subsampled test data, extender test data, and single‐study data). Figure S2: Four‐week remission: BAC for glmnet (subsampled test data, extender test data, and single‐study data). Figure S3: Four‐week remission: BAC for rf (subsampled test data, extender test data, and single‐study data). Figure S4: Four‐week remission: BAC for treebag (subsampled test data, extender test data, and single‐study data). Figure S5: Four‐week remission: BAC for xgbTree (subsampled test data, extender test data, and single‐study data). [file ACPS-152-441-s001.docx]

**Supporting Information**

*Predicting remission in schizophrenia using machine learning – assessing the impact of sample size and predictor overinclusion*

Hieronymus F, Hieronymus M, Sjöstedt A, Nilsson S, Näslund J, Lisinski A, Østergaard SD

**Appendix.** Description of included trials

**Supplementary methods.**

**Table S1.** Hyperparameter tuning using genetic algorithms and grid search

**Table S2.** Baseline item scores for the test-and-train ­set and the ­extender set.

**Table S3.** Additional metrics for the ensemble model

**Table S4.** Additional metrics for the glm model

**Table S5.** Additional metrics for the glmnet model

**Table S6.** Additional metrics for the rf model

**Table S7.** Additional metrics for the treebag model

**Table S8.** Additional metrics for the xgbTree model

**Figure S1.** Four-week remission: BAC for glm (subsampled test data, extender test data, and single-study data)

**Figure S2.** Four-week remission: BAC for glmnet (subsampled test data, extender test data, and single-study data)

**Figure S3.** Four-week remission: BAC for rf (subsampled test data, extender test data, and single-study data)

**Figure S4.** Four-week remission: BAC for treebag (subsampled test data, extender test data, and single-study data)
 **Figure S5.** Four-week remission: BAC for xgbTree (subsampled test data, extender test data, and single-study data)

**Appendix. Description of included trials**

*Placebo-controlled set:*

#1 – Protocol No.: PALM-JPN-4: A Randomized, Double-Blind, Placebo-Controlled, Parallel Group, Fixed-Dose, Multicenter Study of JNS010 (Paliperidone Palmitate) in Patients With Schizophrenia. NCT01299389.

#2 – Protocol No.: R076477-PSZ-3001: A Randomized, Multicenter, Double-Blind, Weight-Based, Fixed-Dose, Parallel-Group, Placebo-Controlled Study of the Efficacy and Safety of Extended Release Paliperidone for the Treatment of Schizophrenia in Adolescent Subjects, 12 to 17 Years of Age. NCT00518323. [“*Teens”* in Chekroud et al.]

#3 – Protocol No.: R076477-SCA-3001: A Randomized, Double-Blind, Placebo-Controlled, Parallel-Group, Study to Evaluate the Efficacy and Safety of Two Dosages of Paliperidone ER in the Treatment of Subjects with Schizoaffective Disorder. NCT00397033.

#4 – Protocol No.: R076477-SCA-3002: A Randomized, Double-Blind, Placebo-Controlled, Parallel-Group Study to Evaluate the Efficacy and Safety of Flexible Dose Paliperidone ER in the Treatment of Subjects with Schizoaffective Disorder. NCT00412373.

#5 – Protocol No.: R076477-SCH-3015: A Randomized, Double-Blind, Placebo-Controlled, Parallel-Group Study to Evaluate the Efficacy and Safety of Paliperidone ER Compared to Quetiapine in Subjects with an Acute Exacerbation of Schizophrenia. NCT00334126. [“*Adults, first episode”* in Chekroud et al.]

#6 – Protocol No.: R076477-SCH-302: A Randomized, 6-Week Double-Blind, Placebo-Controlled Study With an Optional 24-Week Open-Label Extension to Evaluate the Safety and Tolerability of Flexible Doses of Extended Release OROS® Paliperidone in the Treatment of Geriatric Subjects With Schizophrenia. NCT00085748. [“*Older adults”* in Chekroud et al.]

#7 – Protocol No.: R076477-SCH-303: A Randomized, Double-Blind, Placebo- and Active-Controlled, Parallel-Group, Dose-Response Study to Evaluate the Efficacy and Safety of 3 Fixed Dosages of Extended Release OROS® Paliperidone (6, 9, and 12 mg/day) and Olanzapine (10 mg/day), With Open-Label Extension, in the Treatment of Subjects With Schizophrenia. NCT00078039. [“*Adults – Chronic #2”* in Chekroud et al.]

#8 – Protocol No.: R076477-SCH-304: A Randomized, Double-Blind, Placebo- and Active-Controlled, Parallel-Group, Dose-Response Study to Evaluate the Efficacy and Safety of 2 Fixed Dosages of Extended Release OROS® Paliperidone (6 and 12 mg/day) and Olanzapine (10 mg/day), With Open-Label Extension, in the Treatment of Subjects With Schizophrenia. NCT00077714.

#9 – Protocol No.: R076477-SCH-305: A Randomized, Double-Blind, Placebo- and Active-Controlled, Parallel-Group, Dose-Response Study to Evaluate the Efficacy and Safety of 3 Fixed Dosages of Extended Release OROS® Paliperidone (3, 9, and 15 mg/day) and Olanzapine (10 mg/day), With Open-Label Extension, in the Treatment of Subjects With Schizophrenia. NCT00083668. [“*Adults – Chronic #1”* in Chekroud et al.]

#10 – Protocol No.: R076477-SCH-4012: A Randomized, Double-Blind, Placebo- and Active-Controlled, Parallel-Group Study to Evaluate the Efficacy and Safety of a Fixed Dosage of 1.5 mg/day of Paliperidone Extended Release (ER) in the Treatment of Subjects With Schizophrenia. NCT00524043.

#11 – Protocol No.: R092670-PSY-3003: A Randomized, Double-Blind, Placebo-Controlled, Parallel-Group, Dose-Response Study to Evaluate the Efficacy and Safety of 3 Fixed Doses (50 mg eq., 100 mg eq., and 150 mg eq.) of Paliperidone Palmitate in Subjects With Schizophrenia. NCT00210548.

#12 – Protocol No.: R092670-PSY-3004: A Randomized, Double-Blind, Placebo-Controlled, Parallel-Group, Dose-Response Study to Evaluate the Efficacy and Safety of 3 Fixed Doses (25 mg eq., 50 mg eq., and 100 mg eq.) of Paliperidone Palmitate in Subjects With Schizophrenia. NCT00101634.

#13 – Protocol No.: R092670-PSY-3007: A Randomized, Double-Blind, Placebo-Controlled, Parallel-Group, Dose-Response Study to Evaluate the Efficacy and Safety of 3 Fixed Doses (25 mg eq., 100 mg eq., and 150 mg eq.) of Paliperidone Palmitate in Subjects With Schizophrenia. NCT00590577.

#14 – Protocol No.: R092670-SCH-201: A Randomized, Double-Blind, Placebo-Controlled Study to Evaluate the Efficacy and Safety of 50 and 100 mg eq. of Paliperidone Palmitate in Subjects With Schizophrenia. NCT00074477.

#15 – Protocol No.: RIS-INT-3: A randomized, double-blind, placebo-controlled multicenter study compared four fixed doses of risperidone and one dose of haloperidol in schizophrenic patients. Patients were treated for eight weeks with risperidone 2, 6, 10, or 16 mg, haloperidol 20 mg, or placebo. NCT00249132.

#16 – Protocol No.: RIS-SCH-302: A Randomized, Double-Blind, Placebo-Controlled Clinical Study of the Efficacy and Safety of Risperidone for the Treatment of Schizophrenia in Adolescents. NCT00088075.

#17 – Protocol No.: RIS-USA-121: Risperidone depot (microspheres) vs. placebo in the treatment of patients with schizophrenia. NCT00253136.

#18 – Protocol No.: RIS-USA-72: The Safety and Effectiveness of Risperidone 8 mg QD and 4 mg QD Compared to Placebo in the Treatment of Schizophrenia. NCT unavailable.

*Active-controlled set:*

Protocol No.: R092670-PSY-3002: A Randomized, Double Blind, Parallel-Group Comparative Study of Flexibly Dosed Paliperidone Palmitate (25, 50, 75, or 100 mg eq.) Administered Every 4 Weeks and Flexibly Dosed RISPERDAL® CONSTA® (25, 37.5, or 50 mg) Administered Every 2 Weeks in Subjects With Schizophrenia. NCT00210717.

Protocol No.: R076477-PSZ-3003: A Randomized, Multicenter, Double-Blind, Active-Controlled, Flexible-Dose, Parallel-Group Study of the Efficacy and Safety of Extended Release Paliperidone for the Treatment of Symptoms of Schizophrenia in Adolescent Subjects, 12 to 17 Years of Age. NCT01009047.

Protocol No.: RIS-USA-231: The Efficacy and Safety of Risperidone in the Treatment of Adolescents with Schizophrenia. NCT00034749.

**Supplementary methods**

*Outcomes*

For symptomatic remission to be at hand a patient must have a Positive and Negative Syndrome Scale (PANSS) score of no more than three on three positive symptoms (P01 Delusions, P02 Conceptual disorganization and P03 Hallucinations), three negative symptoms (N01 Blunted affect, N04 Passive/apathetic social withdrawal and N06 Lack of spontaneity and flow of conversation) and two general symptoms (G05 Mannerisms and posturing and G09 Unusual thought content).

*Data imputation*

The dataset used by Chekroud et al. consisted of four-week completer data (with some data imputation) for 1513 patients spread across five placebo-controlled trials (n=99, 182, 321, 430, 481). Our placebo-controlled set consists of 4634 patients with completer data from eighteen placebo-controlled trials. Since there was very little missing data for the predictors that we included (~0.5% across all predictors combined), we did not conduct any data imputation and instead dropped the 25 cases for which we lacked data on at least one predictor.

*Assessing the impact of a high number of uninformative predictors on model performance*

To assess the impact of including a high number of predictors, we focused our analyses on 33 variables: age, sex, treatment (antipsychotic or placebo) and baseline scores on the 30 symptom items of the PANSS scale. These 33 predictors were all included in the analysis by Chekroud and co-workers. We then constructed a second predictor set which included those 33 predictors, as well as several simulated predictors known to be uninformative (i.e., uncorrelated to the outcome studied). This was done to assess the models’ ability to parse out uninformative data. To facilitate comparison, we included as many uninformative predictors as the difference between our 33 predictors and the predictor sets used by Chekroud et al. (p=184 and 104, for elastic net and random forest models, respectively). To resemble the different types of data that can be encountered in a clinical data set, noise variables were simulated with one quarter of the variables each having an exponential, normal, Poisson (lambda = 5) and uniform distribution, respectively. Uninformative variables were simulated using the corresponding built-in R functions (rexp, rnorm, rpois and runif).

To estimate the variability inherent in the data we used Monte Carlo subsampling from the placebo-controlled trials to construct training sets of the same five sizes as used to estimate leave-one-study-out BAC in the analysis by Chekroud and co-workers (i.e., 1513 minus the number of patients in each holdout trial). Subsampling was done 50 times for each trial for the elastic net model and, due to processing power limitations, 25 times for each trial for the random forest models. As in Chekroud et al, each elastic net model was trained using 400 combinations of alpha and lambda values according to carets built in-tuning algorithm, and each random forest model on 20 different mtry values also using caret’s prespecified tunings. Caret’s preprocessing function was used to center and scale all variables, as well as to remove zero- and near zero-variance variables.

For each subsampled training set we then used Monte Carlo subsampling to randomly draw a test set of equal size to the corresponding holdout trial (i.e., so that each training and test set size added up to 1513 patients) from the cases not selected for training. The fitted models were used to predict symptomatic remission in the test set. This process was repeated 100 times for each Monte Carlo subsampling using non-parametric bootstrapping. In total, each out-sample balanced accuracy estimate is thus based on 5000 combinations of subsampled training sets and subsampled and bootstrapped test sets for the elastic net models, and 2500 such combinations for random forest models. These results were used to construct 95% CIs for BAC, which were contrasted to the leave-one-study-out estimates reported by Chekroud et al.

*Hyperparameter tuning*

The glm and treebag models do not require hyperparameter tuning. The rf model has a finite hyperparameter space where all possible parameters can be explored using grid search. Glmnet and xgbTree have infinite hyperparameter spaces which can only be partially explored. Using the full placebo-controlled set, we used an exhaustive grid search to find the best hyperparameters for the rf algorithm, and we sought for the optimal hyperparameter tunings for glmnet and xgbTree using genetic algorithms (R-package: GA). As for all other models, 10-fold cross-validation was used for the models in the GA tuning and all models maximized AUC-ROC. The search bounds for the genetic algorithm were iteratively tightened, first after an initial run of ten generations, and then after sequential runs of 100 generations in cases where there was still improvement occurring at least every 30th generation. The best solution from each run was included as a suggestion in subsequent runs. For all runs, the mutation rate was set to 0.25 and the crossover probability to 0.80, with a population size of 100. Local optimization was used initially, but since it was very resource intensive and did not seem to result in additional improvement, it was only used for the initial runs. Tuning parameters as well as the outcomes for all searches are detailed in Supplementary table 1. We also tried Bayesian Optimization (R-package: rBayesianOptimization), but this did not yield better results (data not shown).

*Analyses of simulated data*

We assessed the performance of logistic regression, and an elastic net model trained using 400 built-in hyperparameter suggestions, on simulated data where predictor strength and the ratio of informative to uninformative variables could be controlled.

For strong predictors (r=0.20, 0.35, 0.50), simulated datasets ranged in size from 250 to 5000 cases (250, 500, 750, 1000, 2500, 5000). For weak predictors (r=0.05, 0.10, 0.15) datasets ranged in size from 2000 to 25000 cases (2000, 3000, 4000, 5000, 7500, 10000, 15000, 25000). Datasets were split 80:20 into training and testing sets. Cases were simulated by a vector, Y, of random normal variables (µ = 0, σ = 1) with cases defined as Y > 0. For strong predictors, each dataset included 10 informative predictors while for weak predictors each dataset included 50 informative predictors. Predictors were generated using the formula:

$$X_{i}= rY+\sqrt{1-r^{2}}\cdot Z_{i}$$

where Y is the case variable vector and Z_i_ are independent random normal vectors with µ = 0 and σ = 1. Each data set also included a number (p=90 for strong predictors; p=150 or 450 for weak predictors) of random normal noise variables (µ = 0, σ = 1); corresponding to 1:3 and 1:9 ratios of informative to uninformative variables. All simulations were repeated 50 times in order to get stable estimates.

In total we thus simulated 900 (6 · 3 · 50) datasets of different sizes and with different predictor strengths for strong predictors, and 2400 (8 · 3 · 2 · 50) datasets for weak predictors. For weak predictors we also varied the ratio of informative to uninformative variables. Model performance was compared to the balanced accuracy (BAC) given by the sum (W) of all n informative predictors (i.e., disregarding all uninformative variables):

$$W= X_{1}+X_{2}+\ldots X_{n}$$

If we use W > 0 as cutoff to maximize BAC and then condition on case status Y and define W_+_ = W|Y > 0 and W_−_ = W|Y < 0, then the expectation, variance and sensitivity are

$$\mu W_{+}=E\left( W_{+} \right)=nr\sqrt{2/\pi}$$

$$\sigma_{W_{+}}^{2}=Var\left( W_{+} \right)=n^{2}r^{2}\left( 1-\frac{2}{\pi} \right)+n(1-r^{2})$$

$$sensitivity=P(W>0|Y>0)\approx\Phi(\frac{\mu W_{+}}{\sigma_{W_{+}}})$$

where $\Phi$ is the cumulative distribution function of a standardized normal distribution. By symmetry, $\mu W_{-}=-\mu W_{+}{, \sigma}_{W_{+}}^{2}=\sigma_{W_{-}}^{2}$ and $specificity=sensitivity$, thus:

$$BAC=\frac{sensitivity+specificity}{2}\approx\Phi(\frac{\mu W_{+}}{\sigma_{W_{+}}})$$

**Table S1. Hyperparameter tuning using genetic algorithms and grid search**

| **Model** | **Run** | **Max iterations** | **Elitism** | **Bounds** | **Best tuning** | **Outcome** |
| --- | --- | --- | --- | --- | --- | --- |
| glmnet | 1 | 10 | 2 | alpha: 0 to 1  lambda: 0 to 0.2 | alpha: 0.0218092  lambda: 0.0569022 | AUC-ROC = 70.66% |
| glmnet | 2 | 100 | 5 | alpha: 0 to 0.060  lambda: 0 to 0.100 | alpha: 0.0205956  lambda: 0.0669554 | AUC-ROC = 70.74%  Stopped after 84 iterations |
| xgbTree | 1 | 10 | 2 | eta: 0.01 to 0.15  depth: 2 to 8 colsample_bytree: 0.2 to 0.8 subsample: 0.2 to 0.8  nrounds: 100 to 700  gamma = 0  min_child_weight = 5 | eta: 0.0213504  depth: 5 colsample_bytree: 0.343392  subsample: 0.560772  nrounds: 273  gamma = 0  min_child_weight = 5 | AUC-ROC = 73.66% |
| xgbTree | 2 | 100 | 5 | eta: 0.01 to 0.04  depth: 2 to 5 colsample_bytree: 0.3 to 0.7 subsample: 0.3 to 0.7  nrounds: 250 to 400  gamma = 0  min_child_weight = 5 | eta: 0.0207611  depth: 5 colsample_bytree: 0.347036  subsample: 0.504784  nrounds: 348  gamma = 0  min_child_weight = 5 | AUC-ROC = 73.85%  Stopped after 44 iterations |
| rf | N/A | N/A | N/A | mtry=2 to 33 | mtry=3 | AUC-ROC = 73.62%  Grid search |

*The final values reported under “Best tuning” for each model are the hyperparameters used in all ensemble models.*

**Table S2. Baseline item scores for the placebo-controlled ­set and the active-controlled set.**

|  | **Train-and-test set** | | **Extender set** | |
| --- | --- | --- | --- | --- |
| **Item** | **Mean** | **SD** | **Mean** | **SD** |
| P1: Delusions | 4.06 | 1.17 | 3.61 | 1.09 |
| P2: Conceptual disorganization | 3.53 | 1.16 | 3.38 | 0.99 |
| P3: Hallucinations | 3.61 | 1.43 | 3.12 | 1.39 |
| P4: Excitement | 2.85 | 1.21 | 2.54 | 1.11 |
| P5: Grandiosity | 2.38 | 1.39 | 2.00 | 1.11 |
| P6: Suspiciousness/persecution | 3.84 | 1.18 | 3.56 | 1.09 |
| P7: Hostility | 2.46 | 1.24 | 2.13 | 1.05 |
| N1: Blunted affect | 3.40 | 1.18 | 3.47 | 1.04 |
| N2: Emotional withdrawal | 3.54 | 1.06 | 3.50 | 0.93 |
| N3: Poor rapport | 2.97 | 1.13 | 3.01 | 1.03 |
| N4: Passive/apathetic social withdrawal | 3.56 | 1.14 | 3.56 | 1.05 |
| N5: Difficulty in abstract thinking | 3.80 | 1.20 | 3.48 | 1.07 |
| N6: Lack of spontaneity and  flow of conversation | 3.06 | 1.26 | 3.11 | 1.12 |
| N7: Stereotyped thinking | 3.12 | 1.11 | 3.04 | 1.01 |
| G1: Somatic concern | 2.52 | 1.24 | 2.29 | 1.14 |
| G2: Anxiety | 3.16 | 1.11 | 2.99 | 1.02 |
| G3: Guilt feelings | 2.14 | 1.23 | 1.88 | 1.01 |
| G4: Tension | 3.07 | 1.09 | 2.88 | 0.92 |
| G5: Mannerisms and posturing | 2.47 | 1.16 | 2.38 | 1.13 |
| G6: Depression | 2.57 | 1.28 | 2.33 | 1.11 |
| G7: Motor retardation | 2.35 | 1.15 | 2.37 | 1.09 |
| G8: Uncooperativeness | 2.29 | 1.20 | 2.10 | 1.10 |
| G9: Unusual thought content | 3.50 | 1.18 | 3.12 | 1.12 |
| G10: Disorientation | 2.02 | 1.07 | 1.88 | 0.95 |
| G11: Poor attention | 2.94 | 1.09 | 3.00 | 0.93 |
| G12: Lack of judgment and insight | 3.69 | 1.16 | 3.47 | 1.03 |
| G13: Disturbance of volition | 3.03 | 1.08 | 3.09 | 1.02 |
| G14: Poor impulse control | 2.53 | 1.18 | 2.26 | 1.09 |
| G15: Preoccupation | 3.32 | 1.11 | 3.14 | 0.97 |
| G16: Active social avoidance | 3.35 | 1.10 | 3.26 | 1.03 |

**Table S3. Additional metrics for the ensemble model**

| **Sample** | **Training set size** | **Train BAC (mean)** | **Train BAC (sd)** | **Test BAC (mean)** | **Test BAC (sd)** | **False positives (mean)** | **True positives (mean)** | **False negatives (mean)** | **True negatives (mean)** | **Sensitivity** | **Specificity** | **F1** |
| --- | --- | --- | --- | --- | --- | --- | --- | --- | --- | --- | --- | --- |
| **Subsamples** | 384 | 0.599 | 0.034 | 0.599 | 0.033 | 22 | 29 | 59 | 141 | 0.33 | 0.87 | 0.42 |
|  | 884 | 0.616 | 0.019 | 0.618 | 0.030 | 21 | 32 | 56 | 141 | 0.36 | 0.87 | 0.45 |
|  | 1384 | 0.627 | 0.014 | 0.628 | 0.029 | 21 | 34 | 54 | 141 | 0.39 | 0.87 | 0.48 |
|  | 2384 | 0.634 | 0.010 | 0.635 | 0.030 | 22 | 35 | 52 | 141 | 0.40 | 0.87 | 0.49 |
|  | 3384 | 0.637 | 0.007 | 0.638 | 0.028 | 21 | 36 | 52 | 141 | 0.41 | 0.87 | 0.50 |
|  | 4384 | 0.640 | 0.003 | 0.640 | 0.039 | 22 | 37 | 52 | 139 | 0.42 | 0.86 | 0.50 |
| **Extender** | 384 | N/A | N/A | 0.628 | 0.030 | 146 | 268 | 355 | 694 | 0.43 | 0.83 | 0.52 |
|  | 884 | N/A | N/A | 0.656 | 0.018 | 164 | 316 | 307 | 676 | 0.51 | 0.80 | 0.57 |
|  | 1384 | N/A | N/A | 0.661 | 0.018 | 164 | 323 | 301 | 675 | 0.52 | 0.80 | 0.58 |
|  | 2384 | N/A | N/A | 0.668 | 0.014 | 170 | 335 | 288 | 671 | 0.54 | 0.80 | 0.59 |
|  | 3384 | N/A | N/A | 0.667 | 0.014 | 171 | 335 | 288 | 669 | 0.54 | 0.80 | 0.59 |
|  | 4384 | N/A | N/A | 0.668 | 0.013 | 169 | 336 | 288 | 670 | 0.54 | 0.80 | 0.60 |

*The figures are averaged over 50 runs for each train set size and then rounded. The number of true and false positives and negatives may hence not always line up with the size of the test (n=250) and extender (n=1463) sets.*

**Table S4. Additional metrics for the glm model**

| **Sample** | **Training set size** | **Train BAC (mean)** | **Train BAC (sd)** | **Test BAC (mean)** | **Test BAC (sd)** | **False positives (mean)** | **True positives (mean)** | **False negatives (mean)** | **True negatives (mean)** | **Sensitivity** | **Specificity** | **F1** |
| --- | --- | --- | --- | --- | --- | --- | --- | --- | --- | --- | --- | --- |
| **Subsamples** | 384 | 0.586 | 0.029 | 0.589 | 0.032 | 31 | 33 | 55 | 131 | 0.38 | 0.81 | 0.43 |
|  | 884 | 0.600 | 0.023 | 0.601 | 0.029 | 25 | 31 | 57 | 137 | 0.35 | 0.85 | 0.43 |
|  | 1384 | 0.605 | 0.014 | 0.605 | 0.029 | 23 | 31 | 57 | 139 | 0.35 | 0.86 | 0.44 |
|  | 2384 | 0.607 | 0.012 | 0.608 | 0.029 | 22 | 31 | 57 | 140 | 0.35 | 0.86 | 0.44 |
|  | 3384 | 0.609 | 0.006 | 0.607 | 0.028 | 21 | 30 | 58 | 141 | 0.34 | 0.87 | 0.43 |
|  | 4384 | 0.610 | 0.003 | 0.605 | 0.040 | 21 | 30 | 59 | 140 | 0.34 | 0.87 | 0.43 |
| **Extender** | 384 | N/A | N/A | 0.608 | 0.026 | 229 | 304 | 319 | 611 | 0.49 | 0.73 | 0.53 |
|  | 884 | N/A | N/A | 0.631 | 0.023 | 205 | 315 | 307 | 635 | 0.51 | 0.76 | 0.55 |
|  | 1384 | N/A | N/A | 0.633 | 0.020 | 187 | 304 | 319 | 653 | 0.49 | 0.78 | 0.55 |
|  | 2384 | N/A | N/A | 0.643 | 0.016 | 182 | 313 | 310 | 658 | 0.50 | 0.78 | 0.56 |
|  | 3384 | N/A | N/A | 0.644 | 0.014 | 179 | 312 | 311 | 661 | 0.50 | 0.79 | 0.56 |
|  | 4384 | N/A | N/A | 0.644 | 0.013 | 174 | 309 | 315 | 665 | 0.50 | 0.79 | 0.56 |

*The figures are averaged over 50 runs for each train set size and then rounded. The number of true and false positives and negatives may hence not always line up with the size of the test (n=250) and extender (n=1463) sets.*

**Table S5. Additional metrics for the glmnet model**

| **Sample** | **Training set size** | **Train BAC (mean)** | **Train BAC (sd)** | **Test BAC (mean)** | **Test BAC (sd)** | **False positives (mean)** | **True positives (mean)** | **False negatives (mean)** | **True negatives (mean)** | **Sensitivity** | **Specificity** | **F1** |
| --- | --- | --- | --- | --- | --- | --- | --- | --- | --- | --- | --- | --- |
| **Subsamples** | 384 | 0.583 | 0.030 | 0.584 | 0.031 | 22 | 26 | 61 | 140 | 0.30 | 0.86 | 0.39 |
|  | 884 | 0.588 | 0.021 | 0.589 | 0.028 | 18 | 25 | 63 | 144 | 0.28 | 0.89 | 0.38 |
|  | 1384 | 0.592 | 0.016 | 0.591 | 0.027 | 17 | 25 | 63 | 145 | 0.28 | 0.90 | 0.38 |
|  | 2384 | 0.593 | 0.012 | 0.593 | 0.027 | 16 | 25 | 62 | 146 | 0.29 | 0.90 | 0.39 |
|  | 3384 | 0.594 | 0.006 | 0.592 | 0.026 | 16 | 25 | 63 | 146 | 0.28 | 0.90 | 0.39 |
|  | 4384 | 0.593 | 0.002 | 0.590 | 0.036 | 16 | 25 | 65 | 145 | 0.28 | 0.90 | 0.38 |
| **Extender** | 384 | N/A | N/A | 0.606 | 0.026 | 155 | 247 | 376 | 685 | 0.40 | 0.82 | 0.48 |
|  | 884 | N/A | N/A | 0.622 | 0.024 | 138 | 255 | 368 | 702 | 0.41 | 0.84 | 0.50 |
|  | 1384 | N/A | N/A | 0.619 | 0.020 | 126 | 242 | 381 | 714 | 0.39 | 0.85 | 0.49 |
|  | 2384 | N/A | N/A | 0.626 | 0.016 | 126 | 251 | 372 | 714 | 0.40 | 0.85 | 0.50 |
|  | 3384 | N/A | N/A | 0.627 | 0.013 | 122 | 248 | 375 | 718 | 0.40 | 0.85 | 0.50 |
|  | 4384 | N/A | N/A | 0.623 | 0.012 | 121 | 243 | 380 | 719 | 0.39 | 0.86 | 0.49 |

*The figures are averaged over 50 runs for each train set size and then rounded. The number of true and false positives and negatives may hence not always line up with the size of the test (n=250) and extender (n=1463) sets.*

**Table S6. Additional metrics for the rf model**

| **Sample** | **Training set size** | **Train BAC (mean)** | **Train BAC (sd)** | **Test BAC (mean)** | **Test BAC (sd)** | **False positives (mean)** | **True positives (mean)** | **False negatives (mean)** | **True negatives (mean)** | **Sensitivity** | **Specificity** | **F1** |
| --- | --- | --- | --- | --- | --- | --- | --- | --- | --- | --- | --- | --- |
| **Subsamples** | 384 | 0.590 | 0.032 | 0.591 | 0.033 | 17 | 25 | 63 | 146 | 0.28 | 0.90 | 0.38 |
|  | 884 | 0.603 | 0.018 | 0.604 | 0.029 | 16 | 27 | 61 | 146 | 0.31 | 0.90 | 0.41 |
|  | 1384 | 0.610 | 0.014 | 0.612 | 0.028 | 16 | 28 | 60 | 146 | 0.32 | 0.90 | 0.42 |
|  | 2384 | 0.617 | 0.010 | 0.618 | 0.028 | 16 | 29 | 58 | 146 | 0.33 | 0.90 | 0.44 |
|  | 3384 | 0.619 | 0.007 | 0.620 | 0.026 | 15 | 29 | 59 | 147 | 0.33 | 0.91 | 0.44 |
|  | 4384 | 0.622 | 0.003 | 0.621 | 0.036 | 16 | 31 | 59 | 144 | 0.34 | 0.90 | 0.45 |
| **Extender** | 384 | N/A | N/A | 0.625 | 0.029 | 117 | 243 | 380 | 723 | 0.39 | 0.86 | 0.49 |
|  | 884 | N/A | N/A | 0.645 | 0.019 | 127 | 275 | 348 | 713 | 0.44 | 0.85 | 0.54 |
|  | 1384 | N/A | N/A | 0.647 | 0.019 | 120 | 272 | 351 | 719 | 0.44 | 0.86 | 0.54 |
|  | 2384 | N/A | N/A | 0.655 | 0.015 | 121 | 283 | 340 | 719 | 0.45 | 0.86 | 0.55 |
|  | 3384 | N/A | N/A | 0.654 | 0.014 | 120 | 281 | 342 | 720 | 0.45 | 0.86 | 0.55 |
|  | 4384 | N/A | N/A | 0.655 | 0.013 | 118 | 282 | 342 | 721 | 0.45 | 0.86 | 0.55 |

*The figures are averaged over 50 runs for each train set size and then rounded. The number of true and false positives and negatives may hence not always line up with the size of the test (n=250) and extender (n=1463) sets.*

**Table S7. Additional metrics for the treebag model**

| **Sample** | **Training set size** | **Train BAC (mean)** | **Train BAC (sd)** | **Test BAC (mean)** | **Test BAC (sd)** | **False positives (mean)** | **True positives (mean)** | **False negatives (mean)** | **True negatives (mean)** | **Sensitivity** | **Specificity** | **F1** |
| --- | --- | --- | --- | --- | --- | --- | --- | --- | --- | --- | --- | --- |
| **Subsamples** | 384 | 0.593 | 0.030 | 0.596 | 0.032 | 31 | 33 | 54 | 132 | 0.38 | 0.81 | 0.44 |
|  | 884 | 0.604 | 0.017 | 0.607 | 0.031 | 28 | 34 | 54 | 134 | 0.39 | 0.83 | 0.45 |
|  | 1384 | 0.613 | 0.013 | 0.613 | 0.030 | 27 | 35 | 53 | 135 | 0.40 | 0.83 | 0.47 |
|  | 2384 | 0.619 | 0.012 | 0.617 | 0.030 | 27 | 35 | 53 | 136 | 0.40 | 0.83 | 0.47 |
|  | 3384 | 0.620 | 0.009 | 0.619 | 0.029 | 26 | 35 | 53 | 136 | 0.40 | 0.84 | 0.47 |
|  | 4384 | 0.621 | 0.005 | 0.625 | 0.040 | 25 | 37 | 53 | 135 | 0.41 | 0.84 | 0.49 |
| **Extender** | 384 | N/A | N/A | 0.621 | 0.027 | 196 | 296 | 327 | 644 | 0.48 | 0.77 | 0.53 |
|  | 884 | N/A | N/A | 0.632 | 0.020 | 197 | 310 | 313 | 643 | 0.50 | 0.77 | 0.55 |
|  | 1384 | N/A | N/A | 0.631 | 0.019 | 188 | 303 | 321 | 652 | 0.49 | 0.78 | 0.54 |
|  | 2384 | N/A | N/A | 0.636 | 0.016 | 194 | 313 | 310 | 646 | 0.50 | 0.77 | 0.55 |
|  | 3384 | N/A | N/A | 0.636 | 0.017 | 190 | 311 | 312 | 649 | 0.50 | 0.77 | 0.55 |
|  | 4384 | N/A | N/A | 0.635 | 0.015 | 186 | 307 | 316 | 653 | 0.49 | 0.78 | 0.55 |

*The figures are averaged over 50 runs for each train set size and then rounded. The number of true and false positives and negatives may hence not always line up with the size of the test (n=250) and extender (n=1463) sets.*

**Table S8. Additional metrics for the xgbTree model**

| **Sample** | **Training set size** | **Train BAC (mean)** | **Train BAC (sd)** | **Test BAC (mean)** | **Test BAC (sd)** | **False positives (mean)** | **True positives (mean)** | **False negatives (mean)** | **True negatives (mean)** | Sensitivity | Specificity | F1 |
| --- | --- | --- | --- | --- | --- | --- | --- | --- | --- | --- | --- | --- |
| **Subsamples** | 384 | 0.601 | 0.035 | 0.605 | 0.033 | 25 | 32 | 56 | 137 | 0.36 | 0.85 | 0.44 |
|  | 884 | 0.616 | 0.015 | 0.619 | 0.030 | 23 | 34 | 54 | 139 | 0.39 | 0.86 | 0.47 |
|  | 1384 | 0.627 | 0.013 | 0.627 | 0.029 | 23 | 35 | 53 | 139 | 0.40 | 0.86 | 0.48 |
|  | 2384 | 0.633 | 0.011 | 0.632 | 0.029 | 22 | 35 | 53 | 140 | 0.40 | 0.86 | 0.48 |
|  | 3384 | 0.635 | 0.007 | 0.636 | 0.028 | 21 | 35 | 53 | 141 | 0.40 | 0.87 | 0.49 |
|  | 4384 | 0.636 | 0.004 | 0.633 | 0.041 | 21 | 36 | 54 | 139 | 0.40 | 0.87 | 0.49 |
| **Extender** | 384 | N/A | N/A | 0.641 | 0.022 | 176 | 306 | 317 | 664 | 0.49 | 0.79 | 0.55 |
|  | 884 | N/A | N/A | 0.653 | 0.018 | 190 | 331 | 292 | 651 | 0.53 | 0.77 | 0.58 |
|  | 1384 | N/A | N/A | 0.657 | 0.018 | 177 | 327 | 297 | 663 | 0.52 | 0.79 | 0.58 |
|  | 2384 | N/A | N/A | 0.662 | 0.015 | 177 | 333 | 290 | 664 | 0.53 | 0.79 | 0.59 |
|  | 3384 | N/A | N/A | 0.664 | 0.015 | 173 | 332 | 291 | 667 | 0.53 | 0.79 | 0.59 |
|  | 4384 | N/A | N/A | 0.665 | 0.013 | 168 | 331 | 293 | 672 | 0.53 | 0.80 | 0.59 |

*The figures are averaged over 50 runs for each train set size and then rounded. The number of true and false positives and negatives may hence not always line up with the size of the test (n=250) and extender (n=1463) sets*

**Figure S1. Four-week remission: BAC for glm (subsampled test data, extender data, and single-study data)

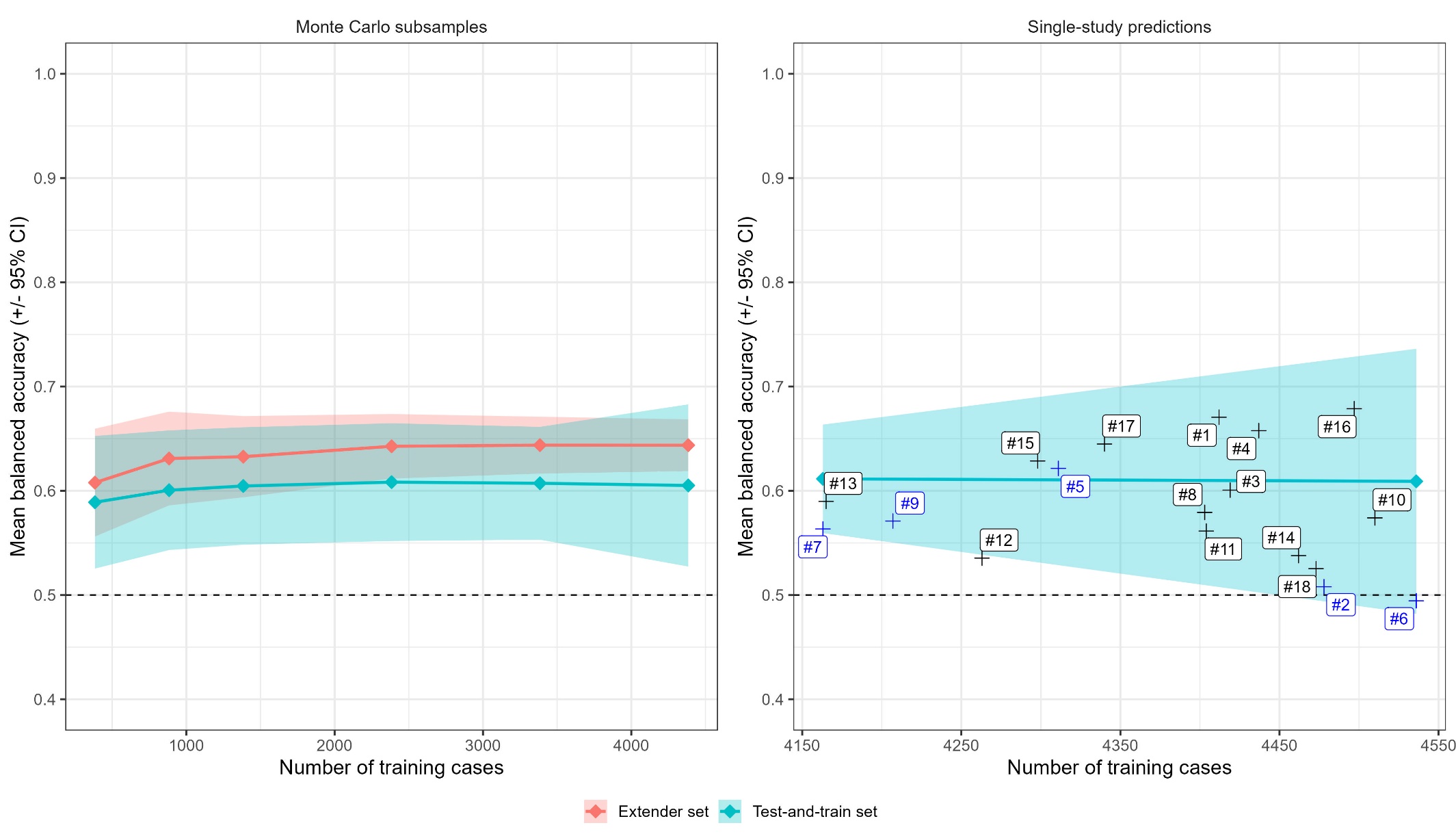
** *The left panel gives mean and 95% CI BAC for subsampled training sets ranging in size between 384 to 4384 cases. The right panel shows the same for subsampled and single-study BAC for leave-one-study-out analyses. Each number #1 to #18 represents one study in the train-and-test set, with blue text indicating a trial included in the analysis by Chekroud and co-workers (see Table 1 for mapping).*

**Figure S2. Four-week remission: BAC for glmnet (subsampled test data, extender data, and single-study data)
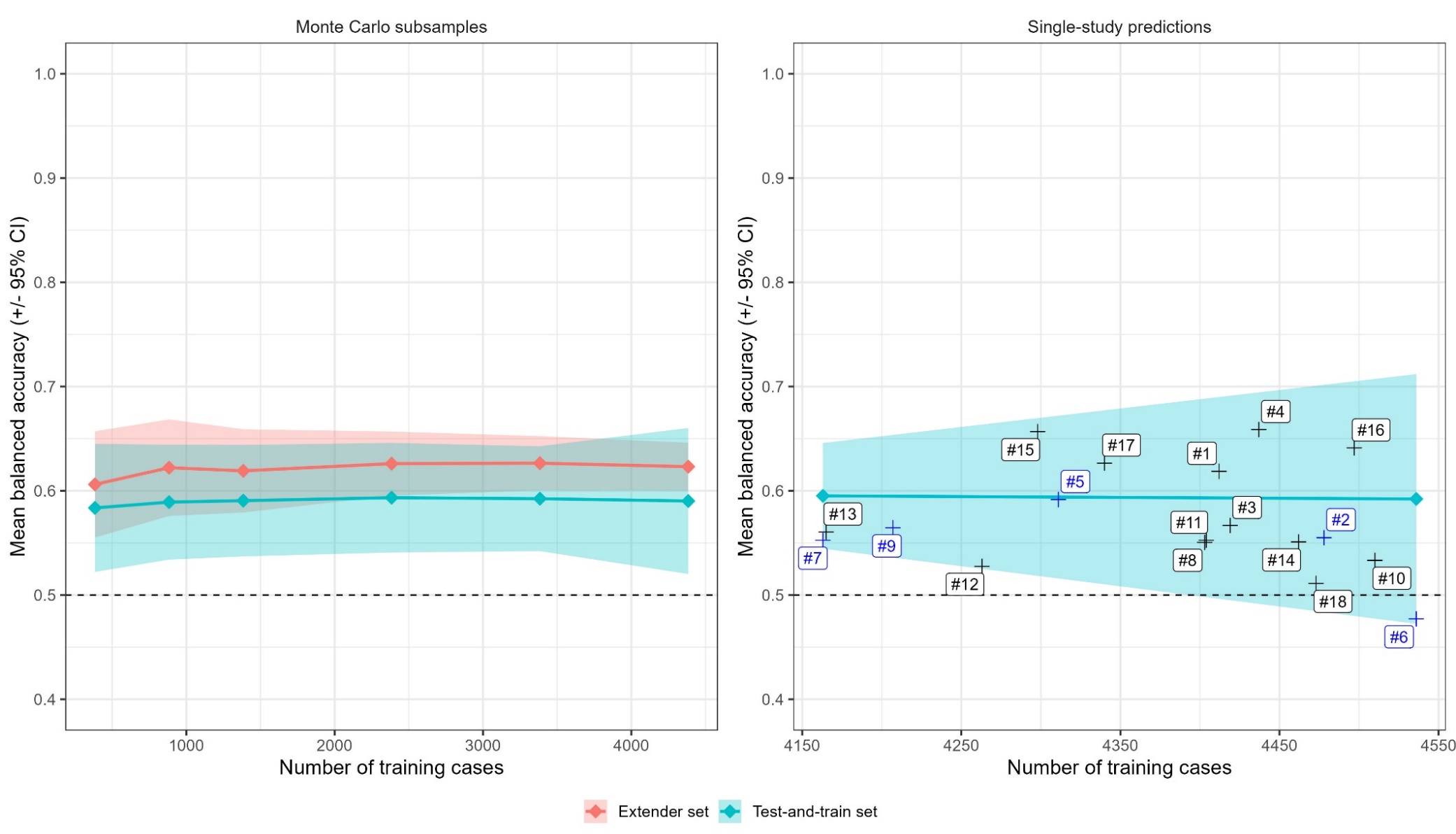
a)***The left panel gives mean and 95% CI BAC for subsampled training sets ranging in size between 384 to 4384 cases. The right panel shows the same for subsampled and single-study BAC for leave-one-study-out analyses. Each number #1 to #18 represents one study in the train-and-test set, with blue text indicating a trial included in the analysis by Chekroud and co-workers (see Table 1 for mapping).*

**Figure S3. Four-week remission: BAC for rf (subsampled test data, extender data, and single-study data)

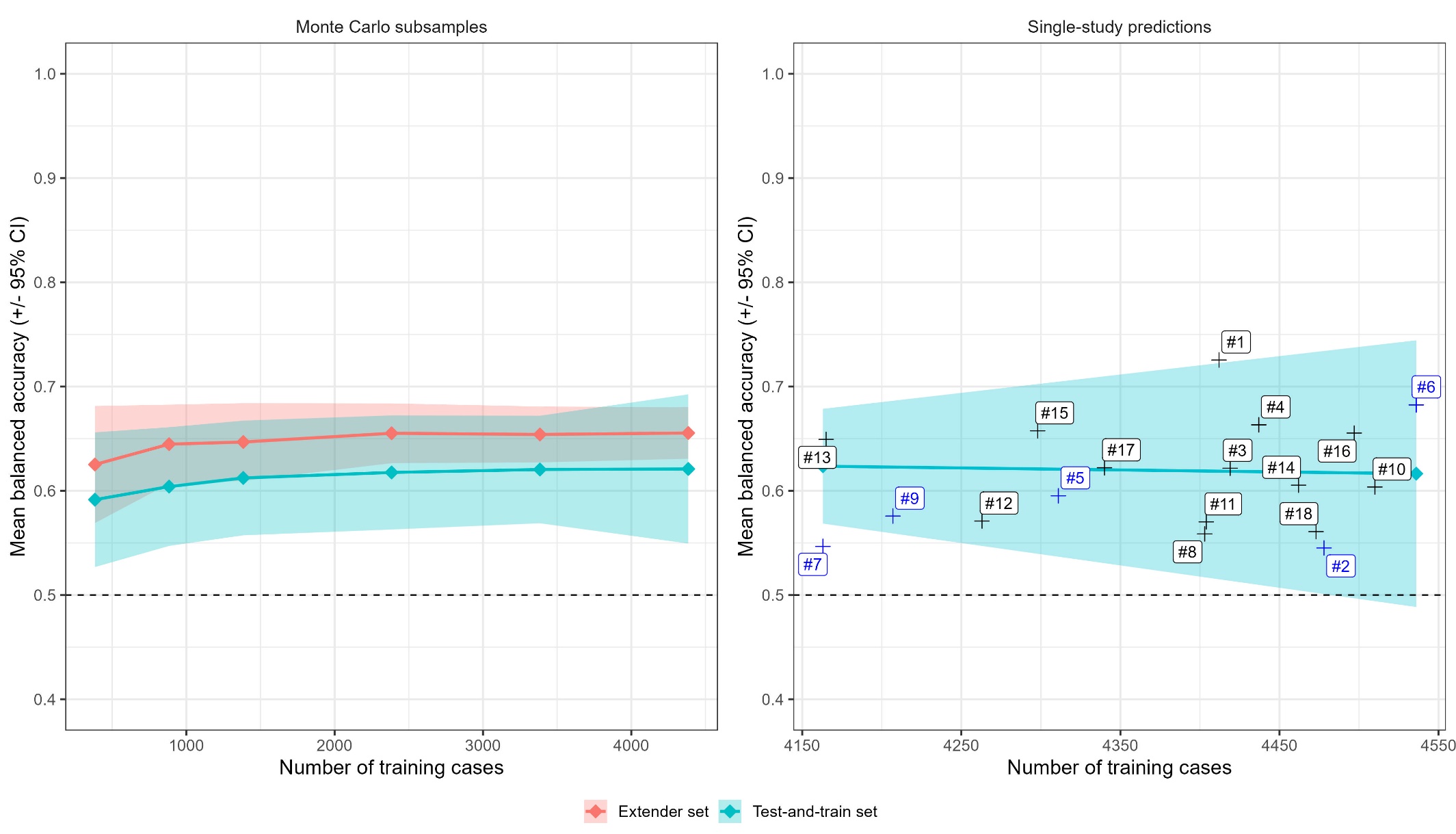
***The left panel gives mean and 95% CI BAC for subsampled training sets ranging in size between 384 to 4384 cases. The right panel shows the same for subsampled and single-study BAC for leave-one-study-out analyses. Each number #1 to #18 represents one study in the train-and-test set, with blue text indicating a trial included in the analysis by Chekroud and co-workers (see Table 1 for mapping).*

**Figure S4. Four-week remission: BAC for treebag (subsampled test data, extender data, and single-study data)
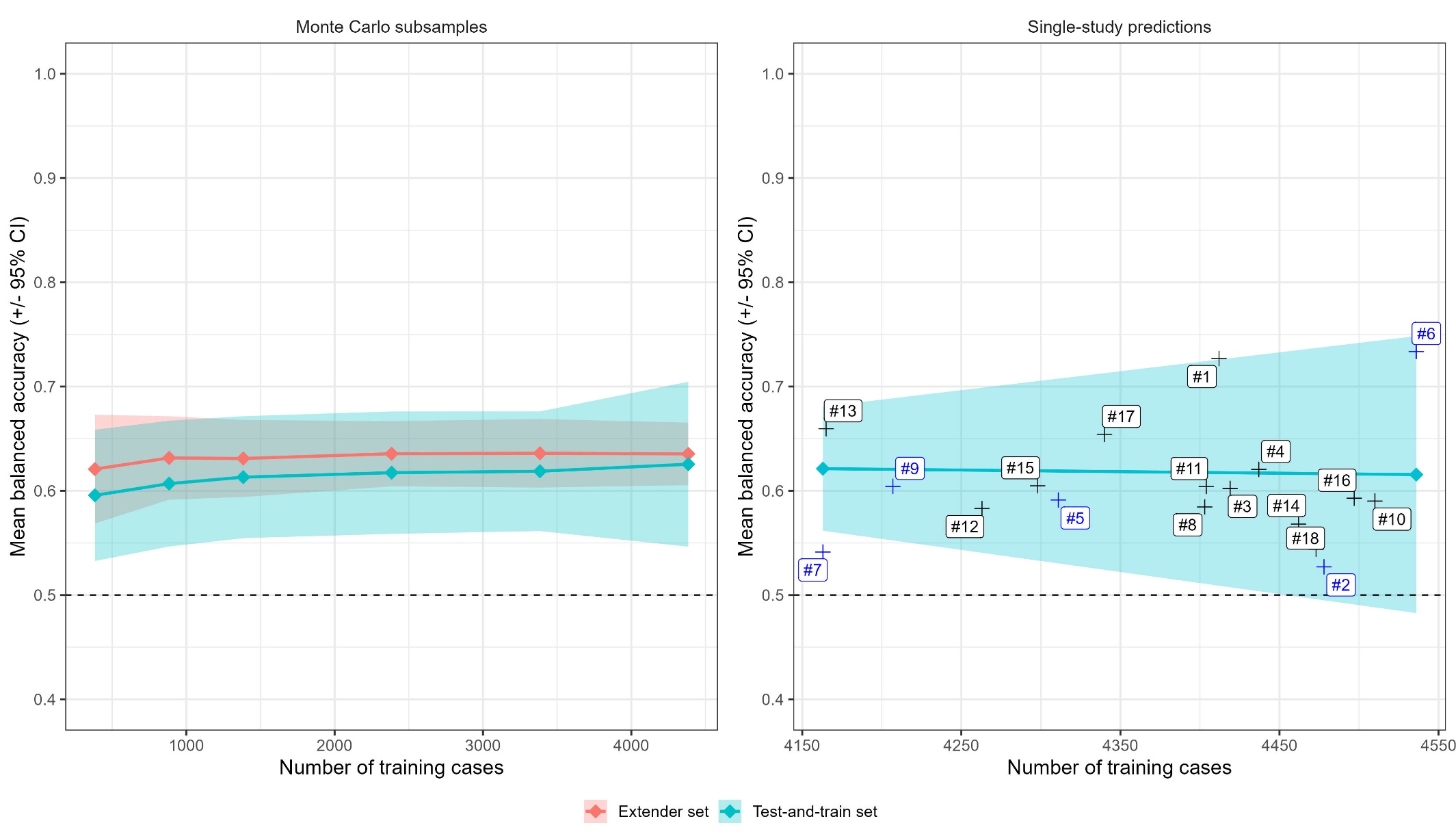
***The left panel gives mean and 95% CI BAC for subsampled training sets ranging in size between 384 to 4384 cases. The right panel shows the same for subsampled and single-study BAC for leave-one-study-out analyses. Each number #1 to #18 represents one study in the train-and-test set, with blue text indicating a trial included in the analysis by Chekroud and co-workers (see Table 1 for mapping).*

**Figure S5. Four-week remission: BAC for xgbTree (subsampled test data, extender data, and single-study data)
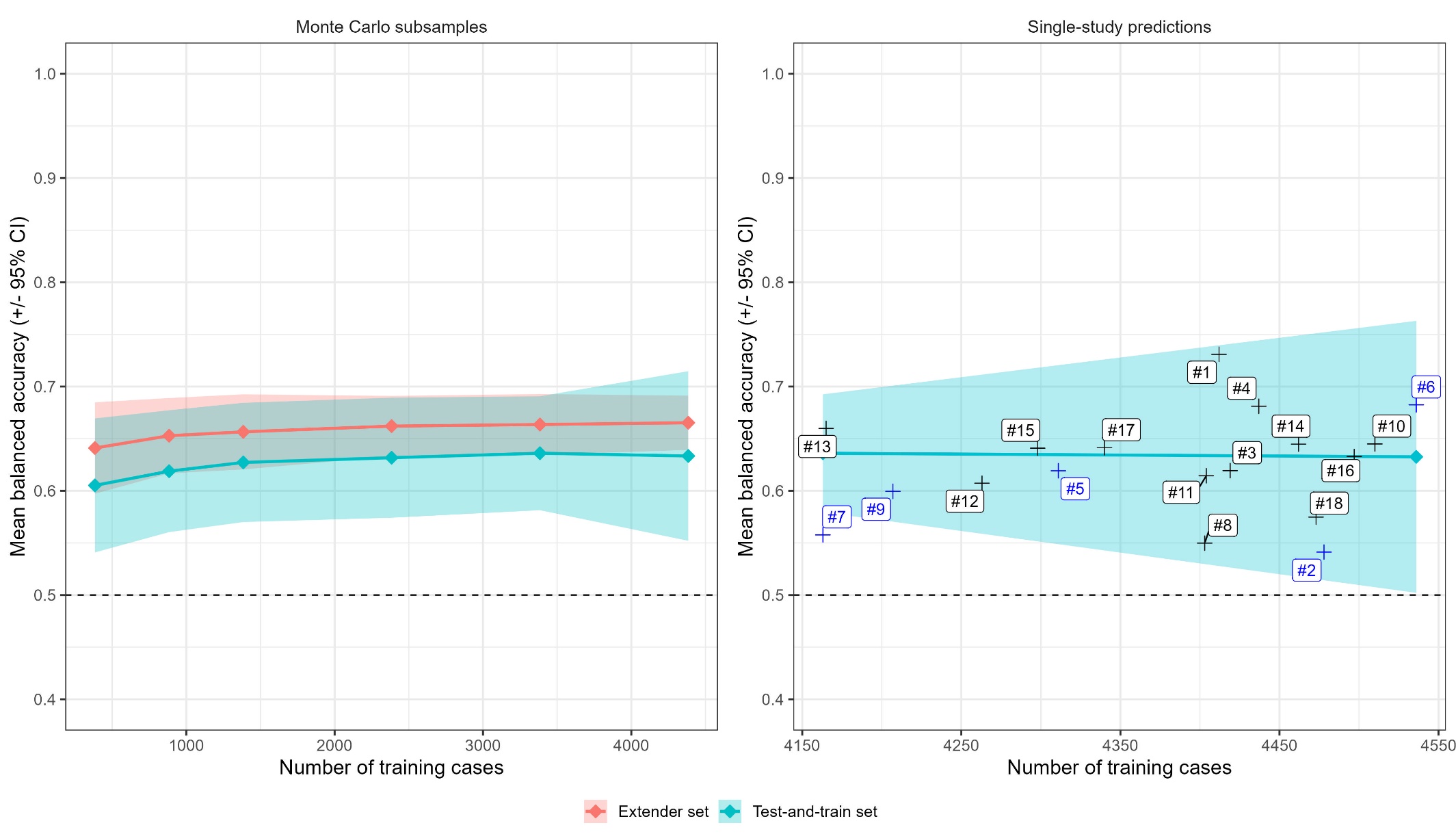
***The left panel gives mean and 95% CI BAC for subsampled training sets ranging in size between 384 to 4384 cases. The right panel shows the same for subsampled and single-study BAC for leave-one-study-out analyses. Each number #1 to #18 represents one study in the train-and-test set, with blue text indicating a trial included in the analysis by Chekroud and co-workers (see Table 1 for mapping).*
